# Catalytic Application and Mechanism Studies of Argentic Chloride Coupled Ag/Au Hollow Heterostructures: Considering the Interface Between Ag/Au Bimetals

**DOI:** 10.1186/s11671-019-2862-9

**Published:** 2019-01-25

**Authors:** Jun Liu, Zhaohui Wu, Quanguo He, Qingyong Tian, Wei Wu, Xiangheng Xiao, Changzhong Jiang

**Affiliations:** 1 Hunan Key Laboratory of Biomedical Nanomaterials and Devices, Hunan University of Technology, Zhuzhou, 412007 People’s Republic of China; 2 Hunan Key Laboratory of Applied Environmental Photocatalysis, Changsha University, Changsha, 410022 People’s Republic of China; 3 Laboratory of Printable Functional Nanomaterials and Printed Electronics, School of Printing and Packaging, Wuhan University, Wuhan, 430072 People’s Republic of China; 4 Key Laboratory of Artificial Micro- and Nano-structures of Ministry of Education, School of Physics and Technology, Wuhan University, Wuhan, 430072 People’s Republic of China

**Keywords:** Ag nanowires, Galvanic replacement reaction, Ag/Au bimetals, Catalysis, Photocatalysis

## Abstract

**Abstract:**

For an economical use of solar energy, photocatalysts that are sufficiently efficient, stable, and capable of harvesting light are required. Composite heterostructures composed of noble metals and semiconductors exhibited the excellent in catalytic application. Here, 1D Ag/Au/AgCl hollow heterostructures are synthesized by galvanic replacement reaction (GRR) from Ag nanowires (NWs). The catalytic properties of these as-obtained Ag/Au/AgCl hollow heterostructures with different ratios are investigated by reducing 4-nitrophenol (Nip) into 4-aminophenol (Amp) in the presence of NaBH_4_, and the influence of AgCl semiconductor to the catalytic performances of Ag/Au bimetals is also investigated. These hollow heterostructures show the higher catalytic properties than pure Ag NWs, and the AgCl not only act as supporting materials, but the excess AgCl is also the obstacle for contact of Ag/Au bimetals with reactive species. Moreover, the photocatalytic performances of these hollow heterostructures are carried out by degradation of acid orange 7 (AO7) under UV and visible light. These Ag/Au/AgCl hollow heterostructures present the higher photocatalytic activities than pure Ag NWs and commercial TiO_2_ (P25), and the Ag/Au bimetals enhance the photocatalytic activity of AgCl semiconductor via the localized surface plasmon resonance (LSPR) and plasmon resonance energy transfer (PRET) mechanisms. The as-synthesized 1D Ag/Au/AgCl hollow heterostructures with multifunction could apply in practical environmental remedy by catalytic manners.

**Graphical abstract:**

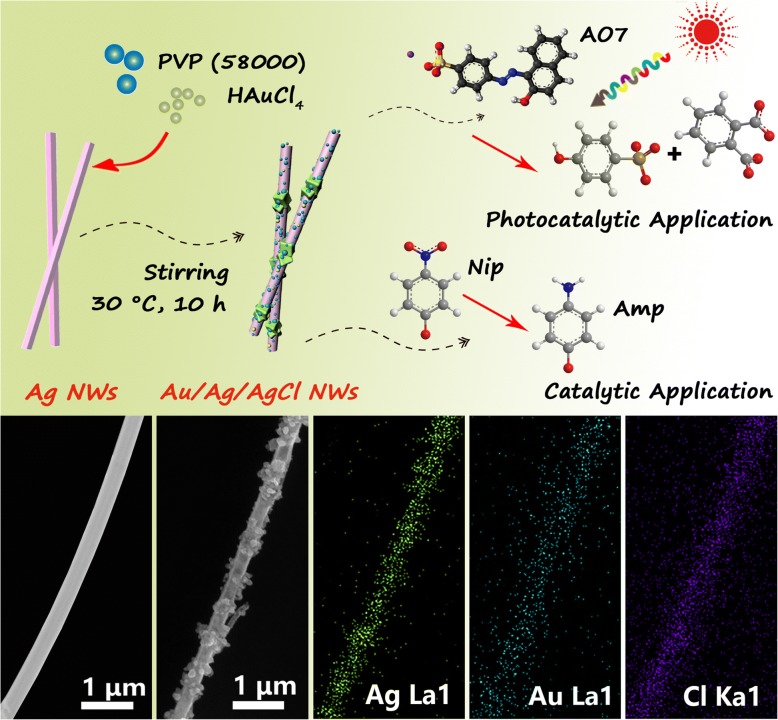

**Electronic supplementary material:**

The online version of this article (10.1186/s11671-019-2862-9) contains supplementary material, which is available to authorized users.

## Background

Noble metal nanoparticles (NPs), including Ag, Au, Pd, and Pt, play an important role in scientific research and national economical construction, because it present good electrical conductivity, chemical reactivity, and photoelectric property for various practical applications [1–3]. Especially, Ag and Au NPs, as typical novel metal materials, with excellent conductivity are frequently used as conducting medium in fuel cell and printed electronics applications, owing to the high stability, resistant to the chemical erosion, antioxidant, and low expansion coefficient [4, 5]. In addition, Ag and Au NPs with high chemical activities could be used in various fields, such as catalysis, light-sensitive apparatus, and biomedicine [6–9]. Under visible light illumination, Ag and Au NPs present the LSPR and PRET effects [10]. It is different from carbon materials coupling with semiconductors [11, 12]; the noble metal coating is not only used for electric conduction, but also for charge produced and transferred by employing these LSPR and PRET effects [13–15].

Recently, bimetal composed of two noble metals are proposed to improve the catalytic performances because of the synergistic effects between two noble metals. As the literature reported, Ag/Au bimetals is the most common bimetal among all noble bimetals, owing to the chemical stability and excellent electron trapping performance [16, 17]. Essentially, the work function of Ag is higher than that of Au, then electrons could transfer from Ag to Au, resulting in the electrons surplus area and electrons depletion area in Au and Ag, respectively. These areas on Au and Ag could increase the chances for absorption of the ions or molecules. Thus, this Au/Ag alloyed bimetal could be used for chemical reaction without light irradiation [18]. For example, as reported by the Yan and co-workers, Au–Ag alloy NPs decorated on GO are synthesized successfully for reduction of 4-nitrophenol to 4-aminophenol. The reaction rate of Au/Ag/GO is 23.26 and 41.15 times higher than that of Au/GO and Ag/GO, respectively [19]. Furthermore, when these bimetals coupling with semiconductors, such as AgCl, the Schottky barrier is formed between the Au/Ag and AgCl. With the assistant of the Schottky barrier, the photo-excited electrons from AgCl semiconductor are effectively transferred to Ag/Au bimetals by comparing with each mono-metal, because the electron depletion area in Ag NPs existed as electron acceptor could improve the separation of electrons [20]. In addition, after absorbing photons, the produced LSPR and PRET effects of Ag/Au bimetals could promote the semiconductors to produce more charges [21, 22]. Based on these theories, inducing Au/Ag bimetal into semi-conductor AgCl is an effective way for improving the photocatalytic performance of AgCl.

Notably, noble metal nanomaterials with various novel shapes, including one-dimensional (1D), two-dimensional (2D), and three-dimensional (3D) morphologies, have shown the fascinating shape-dependent performances in practical application. Particularly, noble metals with multidimensional morphologies possess some advantages over its zero-dimensional (0D) morphology. For example, 1D Ag NWs with large aspect ratio are synthesized by facile hydrothermal method, these Ag NWs present larger surface areas and more excellent electrical conductivity than Ag NPs, providing high potential for high performance flexible transparent electrodes [23]. However, report on 1D Au/Ag bimetal NWs coupling with AgCl for catalytic application are very scarce so far. Herein, 1D Ag/Au/AgCl hollow heterostructures stemmed from Ag NWs are prepared through GRR method. The morphologies of these heterostructures are well controlled by altering the concentration of HAuCl_4_ solution. The shape-dependent catalytic performances of these 1D Ag/Au/AgCl hollow heterostructures for reduction of Nip are investigated. The photocatalytic performances of these samples are also carried out by degradation of AO7. Correspondingly, the shape-dependent proposed catalytic and photocatalytic mechanisms of this hollow heterostructures are discussed.

## Methods

### Materials and Chemicals

Silver nitrate (99.85%, Aladdin Reagents Co., Ltd., Shanghai, China), copper chloride (98.0%, Aladdin Reagents Co., Ltd., Shanghai, China), polyvinylpyrrolidone (MW = 40,000, MW = 58,000, 98.0%, Sigma-Aldrich Co., Shanghai, China), and ethylene glycol (98.0%, Sigma-Aldrich Co., Shanghai, China) were used for synthesis of Ag NWs. Chloroauric acid (99.99%, Alfa-Aesar Co., Shanghai, China) and ethanol (95.0%, Sinopharm Chemical Reagent Co., Ltd., Shanghai, China) were used for synthesis of Ag/Au/AgCl composite NWs. Acid orange 7 (98.0%, Sigma-Aldrich Co., Shanghai, China) and commercial TiO_2_ (P25, 95.0%, Evonik Industries AG., Beijing, China) were used for testing the photocatalytic property. 4-nitrophenol (99.0%, Sinopharm Chemical Reagent Co., Ltd., Shanghai, China) and sodium borohydride (98.0%, Aladdin Reagents Co., Ltd., Shanghai, China) were used for testing the catalytic property. All these reagents were used directly without further treatment.

### Synthesis Processes

#### Synthesized Routes

**Fig. 1:**
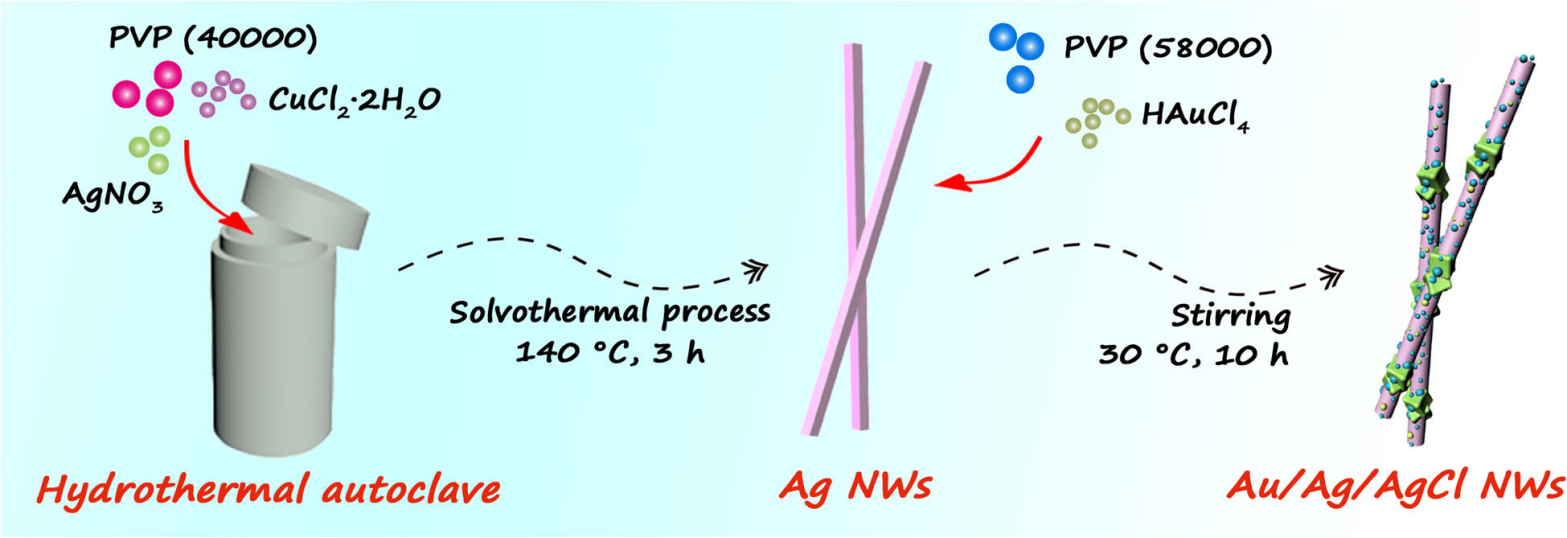
Schematic illustration of the synthetic routes of 1D Ag/Au/AgCl hollow heterostructures

As shown in Fig. 1, two steps (solvothermal method and GRR) are carried out for synthesis of 1D Ag/Au/AgCl hollow heterostructures. Initially, the Ag NWs with uniform morphology are synthesized by solvothermal method with assistance of CuCl_2_ and PVP. Cl^−^ is added for controlling the free Ag^+^ in the initial formation of seeds, and it is facilitated to forming multiply twinned Ag seeds for NWs’ growth. Cu^2+^ is added as a sacrifice agent for eliminating the adsorbed oxygen, because molecular oxygen could absorb on the surface of Ag seeds and prevent the Ag deposition. PVP acts as a capping agent for strong adherent on (100) facet in this reaction, preventing the growth on this facet. While PVP shows weak interaction with the facet of (111); thus, the Ag NWs could grow rapidly on this facet [24, 25]. Thus, the Ag NWs are finally synthesized. Then, the Ag/Au/AgCl hollow heterostructures are synthesized by GRR. The Ag NWs with high-energy sites (such as defect and stacking fault) are processed through GRR after adding HAuCl_4_ solution dropwise [26, 27]. The Au and AgCl precipitates are deposited on the surface of Ag NWs. During this deposition process, the Ag atoms are oxidized into Ag+ and dissolved in solution; a small concave is subsequently formed on the surface of Ag NWs. With the consumption HAuCl_4_ solution, the as-generated Au atoms and AgCl precipitations are gathered on the surrounding of the small hole; thus, the Ag/Au/AgCl hollow heterostructures are formed [28]. The specific chemical reaction formula shows as follows:

$$ 3\mathrm{Ag}+{\mathrm{HAuCl}}_4\to \mathrm{Au}+3\mathrm{Ag}\mathrm{Cl}\downarrow +\mathrm{HCl} $$

#### Synthesis of Ag NWs

The Ag NWs with uniform morphology were synthesized by solvothermal method. Typically, 0.1699 g of AgNO_3_ was dissolved in 10 mL of EG solution under stirring at room temperature. This solution was named as solution A. 0.1665 g of PVP (MW = 40,000) and 0.0019 g of CuCl_2_·2H_2_O were dissolved in 9 mL and 10 mL of EG solution, respectively. Then, 1 mL of the CuCl_2_ EG solution was added into 9 mL of PVP EG solution to form homogeneous solution, named as solution B. Subsequently, the solution B was dropwise added into solution A under stirring at room temperature. This mixture was transferred to 30 mL Teflon-lined stainless-steel autoclave and maintained at 140 °C for 3 h. After cooling down to room temperature, the gray productions were washed by deionized water and ethanol for three times, respectively. These samples were conserved in vacuum.

#### Synthesis of Ag/Au/AgCl Hollow Heterostructures

The Ag/Au/AgCl hollow heterostructures are synthesized by GRR method. Typically, 15 mg of Ag NWs are dispersed in 10 mL of ethanol, 20 mg of PVP (MW = 58,000) were added in under vigorous stirring. Then, 0.5 mL of HAuCl_4_ solution (0.2 Mm) was added in another 9.5 mL of ethanol. Finally, this HAuCl_4_ solution was added in Ag NWs dispersed solution by peristaltic pump at the rate of 0.15 mL min^−1^. This reaction kept at 30 °C for 10 h under continuously stirring. The as-obtained samples were washed by deionized water and ethanol for three times, respectively. The final samples were dried in vacuum oven at 60 °C for 12 h. Other samples (S2 and S3) were obtained by changing the dosage of HAuCl_4_ solution, and the corresponding specific parameters were presented in Table 1.

**Table 1:** Summary of the synthetic condition of samples and the kinetic rate constants (*k*_1_) for catalytic reduction of Nip to Amp, (*k*_2_) for photocatalytic AO7 dye under mix light

Sample	Quality of Ag NWs (mg)	Volume of HAuCl_4_ (mL) (0.2 mM)	Mole ratio of Ag:Au:AgCl (*n*:*n*:*n*)	Catalytic reduction of Nip to Amp		Photodegradation of AO7 dye	
				Used dosage (mg)	*k*_1_ value (10^−2^ min^−1^)	Used dosage (mg)	*k*_*2*_ value (10^−2^ min^−1^)
Bare	/	/	/	/	0.0466	/	1.01
Ag NWs	/	/	/	0.025	0.303	3	1.67
P25	/	/	/	/	/	3	20.5
S1	15	0.5	11:1:3	0.025	123	3	87.9
S2	15	1.0	8:2:6	0.025	177	3	134.0
S2	15	1.0	8:2:6	0.05	512	/	/
S2	15	1.0	8:2:6	0.1	360	/	/
S3	15	1.5	5:3:9	0.025	111	3	89.3

### Characterization

The scanning electron microscopy (SEM) images were photographed by cold field emission SEM (Hitachi S-4800, Japan) at 5 kV. The transmission electron microscopy (TEM) and high-resolution transmission electron microscopy (HRTEM) images were taken by JEOL JEM-2010 (HT, Japan) and operated at 200 kV. The Powder X-ray diffraction (XRD) patterns were measured by PANalytical, (Holland) with Cu Kα radiation, *λ* = 0.1542 nm was used to measure the XRD patterns of these samples, which are operated at 40 kV and 40 mA with scan rate of 0.05° 2θ s^−1^. The XPS spectra were measured by X-ray photoelectron spectroscopy (XPS, Thermo Fisher, USA) with radiation source of Al Kα, 1486.6 eV. The UV-visible absorption spectra of these sample were measured by UV-2550 spectrophotometer (Shimadzu, Japan).

### Catalytic Reduction of 4-Nitrophenol

The catalytic reactions were carried out by reducing 4-nitrophenol (Nip) into 4-aminophenol (Amp). Typically, 150 μL of NaBH_4_ (0.3 M) were added into 4 mL of Nip solution (0.1 mM). Then 0.1 mg of sample (Ag NWs and Ag/Au/AgCl samples) was added into the solution under stirring. Green-yellow solution becomes colorless gradually as the reaction proceeds. The absorption spectra of the solutions were monitored by Shimadzu 2550 UV-visible spectrophotometer (200–500 nm) with a time interval of 60 s at room temperature.

### Photocatalytic Tests

Photocatalytic experiments of as-prepared samples were carried out under simulated sunlight, the simulated sunlight came from metal halide lamp (400 W; intensity 23.13 mW cm^−2^, measured by Newport Power Meter 3936-R). Ten milliliters of AO7 solution (15 mg L^−1^) were added into quartz tube, then, 3 mg of as-obtained samples (Ag NWs and Ag/Au/AgCl samples) were added for experimental groups, and without any sample adding for control group. Before light illumination, the absorption effect of these samples was carried out by stirring in the dark for 30 min. Then, these solutions were illuminated by simulated sunlight with 1 min illumination interval. The absorptions of AO7 were tested by Shimadzu 2550 UV-visible spectrophotometer at the range of 200–800 nm.

## Result and Discussion

### Morphologic Characterization of Ag/Au/AgCl Hollow Heterostructures

**Fig. 2:**
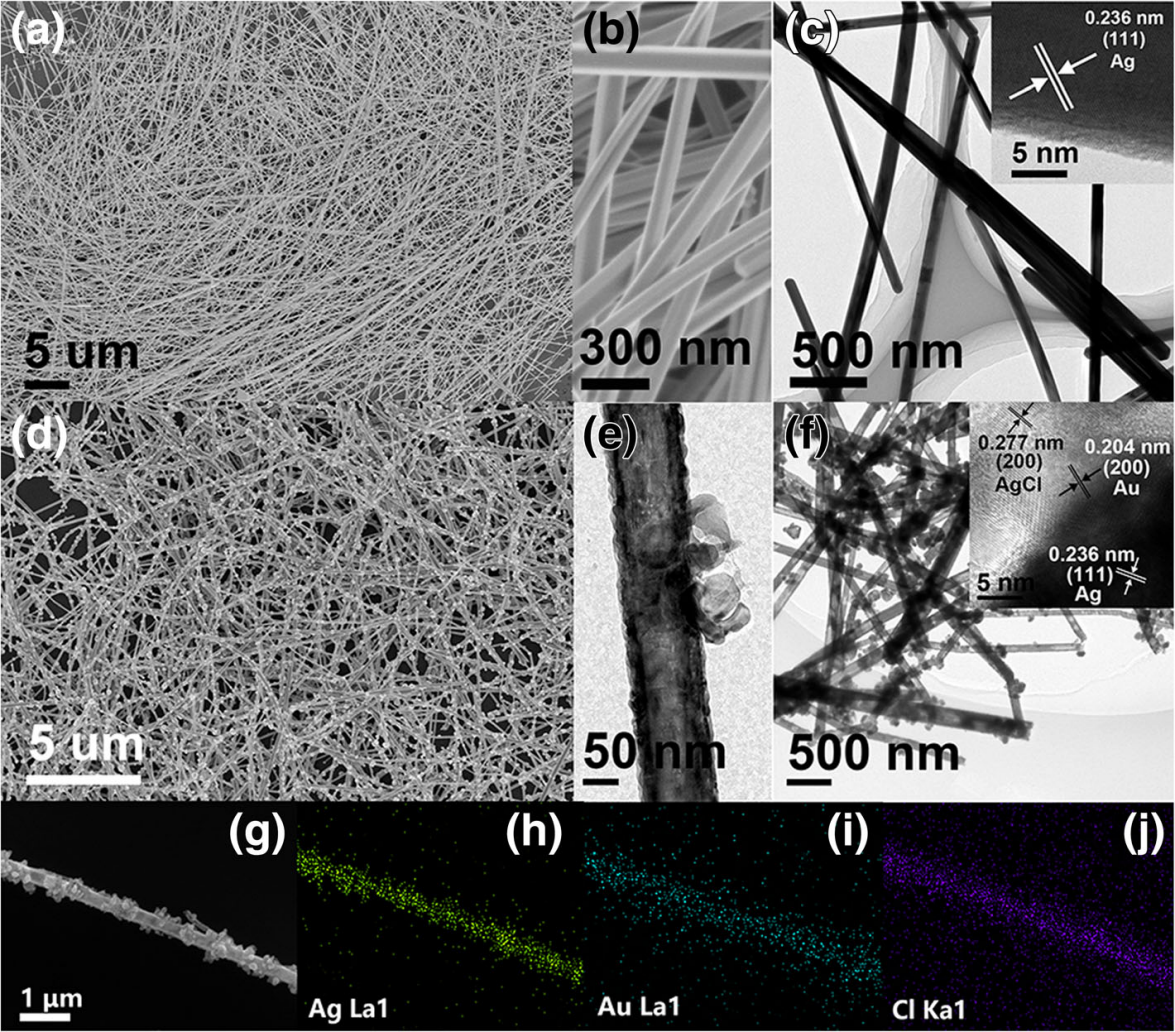
**a** SEM images of Ag NWs. **b** The corresponding magnified SEM image. **c** TEM image of Ag NWs (the inset is HRTEM image of Ag NWs). **d** SEM image of Ag/Au/AgCl hollow heterostructures (S1). **e** The enlarged TEM image of a single Ag/Au/AgCl hollow NWs. **f** TEM image of Ag/Au/AgCl hollow heterostructures (the inset is the HRTEM image of Ag/Au/AgCl hollow heterostructures). **g** SEM image of a single Ag/Au/AgCl NWs. **h–j** The corresponding EDX mapping of Ag, Au, and Cl elements

The morphologies of Ag NWs are characterized by SEM, TEM, and HRTEM (Fig. 2a, b). As shown in Fig. 2a, the large amount of Ag NWs with uniform size and morphology are presented, indicating that the pure Ag NWs without other shapes (such as NPs) are synthesized by this solvothermal method. The average diameter and average length of these Ag NWs are 104 nm and 12 μm, respectively. As shown in the magnified SEM and TEM images, the distinct surface edges of Ag NWs could be observed (Fig. 2b), and the smooth surface of solid Ag NWs is also observed (Fig. 2c). Moreover, the lattice spacing of 0.236 nm could be indexed to (111) facet of metallic Ag (JCPDS No. 04−0783), according to corresponding inserted HRTEM image. Figure 2d shows the SEM image of Ag/Au/AgCl hollow heterostructures. These NPs distribute on the surface of Ag NWs uniformly. Figure 2e, f shows the TEM images of Ag/Au/AgCl hollow NWs. The distinct hollow structure in Ag NWs confirms that the theory of GRR in this synthesis of this heterostructures is feasible. Moreover, the HRTEM image of Ag/Au/AgCl hollow NWs is presented in the inset of Fig. 2f. The lattice spacing of 0.236 and 0.204 nm could be indexed to (111) facet of metallic Ag and (200) facet of metallic Au (JCPDS No. 04–0784), respectively, while the lattice spacing of 0.277 nm belongs to (200) of AgCl (JCPDS No. 22−1326). Meanwhile, the distribution of Ag, Au, and Cl confirms the hollow heterostructure of Ag/Au/AgCl NWs, according to the EDX elemental mapping (Fig. 2g–j). As a result, this Ag/Au/AgCl hollow heterostructures are synthesized successfully.

Additionally, several samples with different ratios (Ag, Au, and AgCl) are prepared. The SEM images of S2 and S3 are presented in Additional file 1: Figure S1a and Figure S1c, respectively. The images in Additional file 1: Figure S1b and Figure S1d are the corresponding enlarged SEM images. With the addition of HAuCl_4_, NPs on the surface of Ag NWs is increased gradually, and the size of them is also increased correspondingly. The NPs are fully coated on the surface of Ag NWs when the dosage of HAuCl_4_ is 1.5 mL (Additional file 1: Figure S1d). Furthermore, the specific parameters for synthesis of samples (Ag NWs and S1-S3) are presented in Table 1. With the increase of HAuCl_4_ solution, Ag NWs are consumed quickly. Theoretically, in S1, S2, and S3, the mole ratio of Ag, Au, and AgCl is 11:1:3, 8:2:6, and 5:3:9, respectively.

### Structural and Elemental Characterization of Ag/Au/AgCl Hollow Heterostructures

**Fig. 3:**
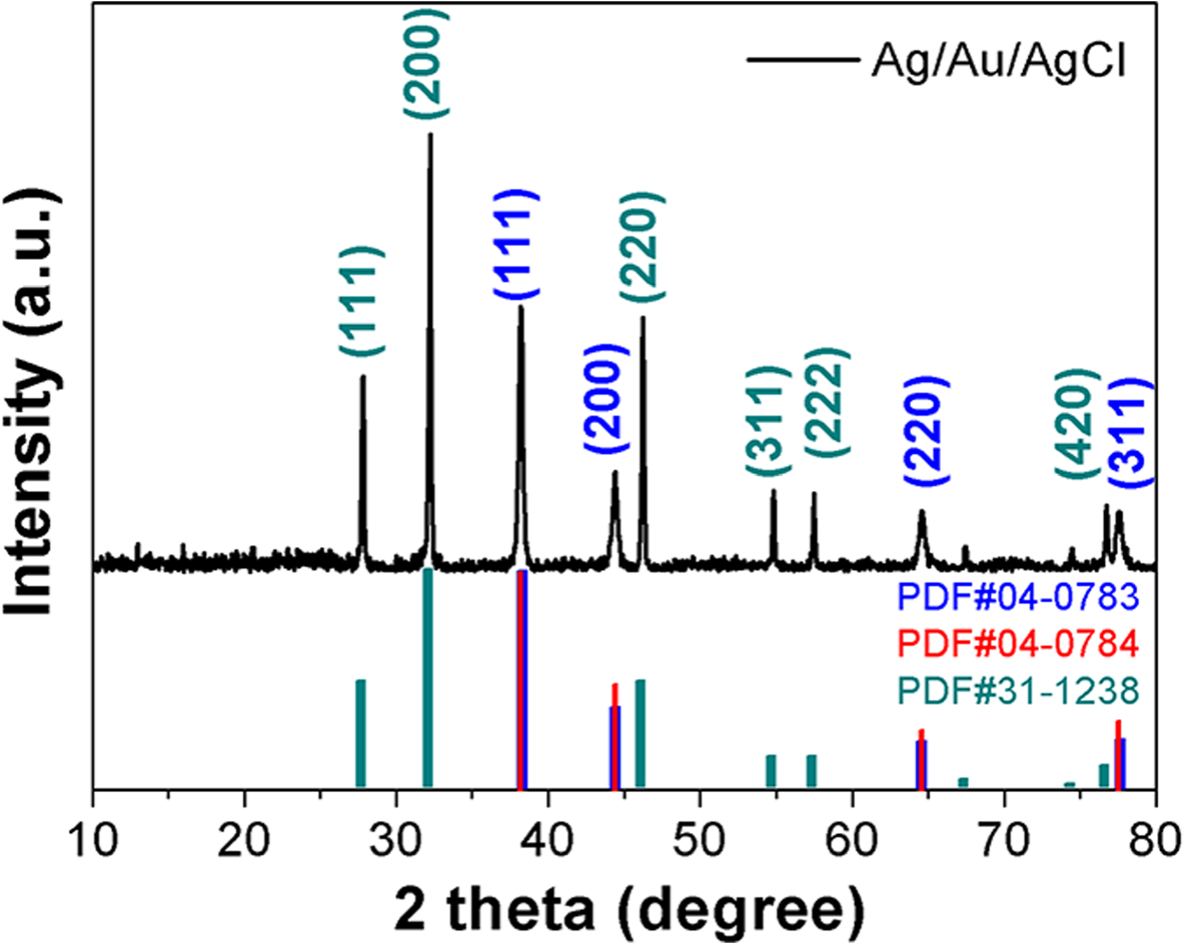
The XRD pattern of as-synthesized Ag/Au/AgCl hollow heterostructure (S1), the standard JCPDS cards of Ag (04−0783, blue column), Au (04−0784, red column), and AgCl (31−1238, green column) are carried out for comparison

The structural characterization of Ag/Au/AgCl hollow heterostructures is carried out by XRD. As shown in this XRD pattern (Fig. 3), the diffraction peaks located at 27.83^°^, 32.24^°^, 46.23^°^, 54.83^°^, 57.48^°^, and 76.73^°^ could be indexed to (111), (200), (220), (311), (222), and (420) facets of chlorargyrite (JCPDS No. 31−1238), respectively. While the diffraction peaks located at 38.11^°^, 44.28^°^, 64.43^°^, and 77.47^°^ belonged to the (111), (200), (220), and (311) of metallic Ag and Au (JCPDS No. 04−0783 and 04−0784). Obviously, the XRD diffraction peak positions of Ag and Au are similar, and the Au could not be distinguished from metallic Ag by this XRD characterization. Thus, the XPS spectra are carried out for further elemental analysis of Ag/Au/AgCl hollow heterostructures.

**Fig. 4:**
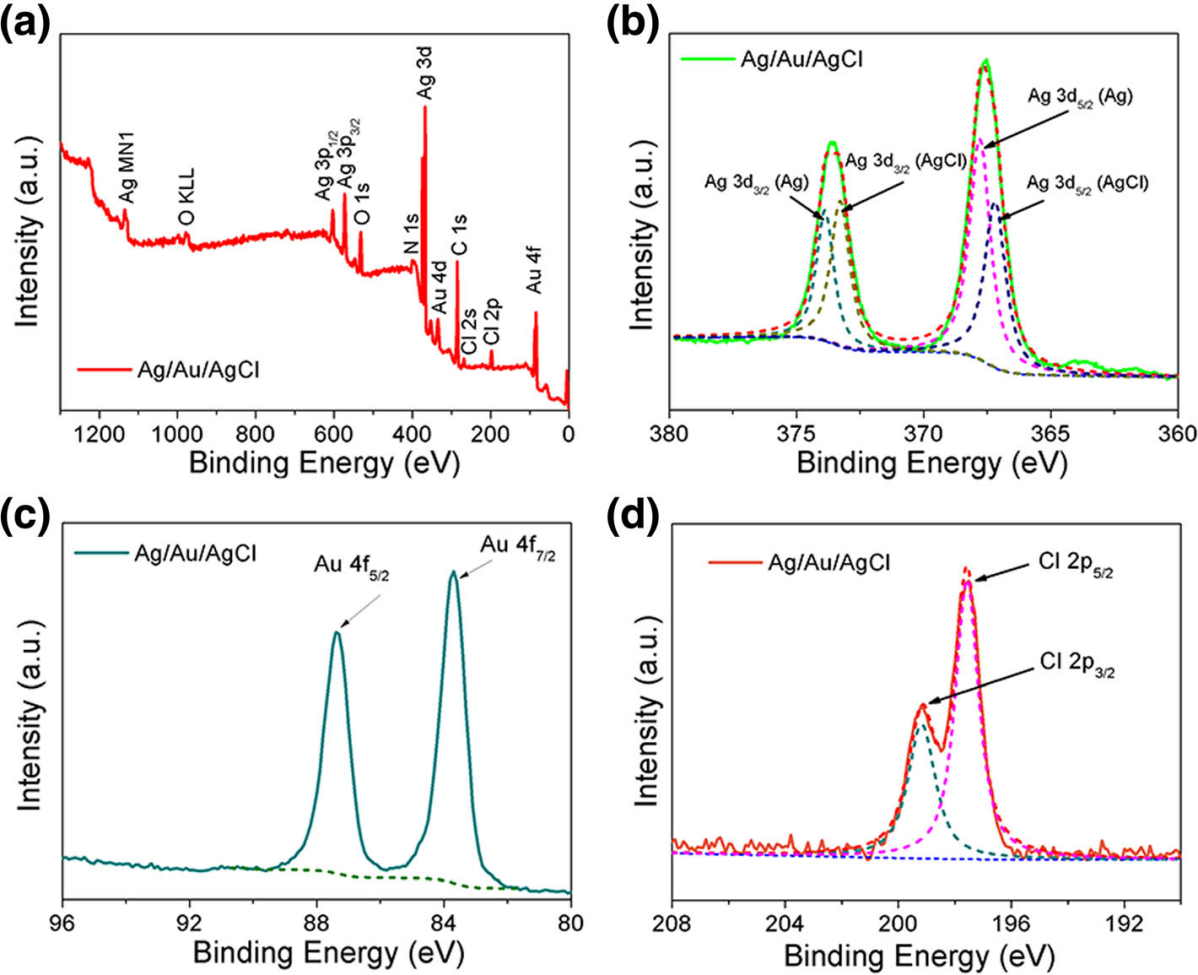
**a** The complete XPS spectra of Ag/Au/AgCl hollow heterostructures. **b** Main peaks of Ag 3d 5/2 and Ag 3d 3/2. **c** Main peaks of Au 4f 7/2 and Au 4f 5/2. **d** Main peaks of Ag 2p 5/2 and Ag 2p 3/2

As shown in Fig. 4a, these elements, including Au, Ag, Cl, O, N, and C, are presented evidently, which means that the Ag, Au, and AgCl are existent in the heterostructure. The high-resolution XPS spectrum of Ag 3d is presented in Fig. 4b; four peaks located at 373.8, 367.8, 373.2, and 367.2 eV are obtained by peak-differentiating technique. Particularly, two peaks located at 373.8 and 367.8 eV are attributed to metallic Ag, and peaks at 373.2 and 367.2 eV belong to AgCl, indicating that both Ag and AgCl are existent in this Ag/Au/AgCl hollow heterostructures simultaneously. In the high-resolution XPS spectrum of Au 4f, the two peaks located at 87.5 and 83.8 eV belong to Au 4f_7/2_ and Au 4f_5/2_ (Fig. 4c). Additionally, the overlapped peaks of Cl 2p_1/2_ and Cl 2p_3/2_ have been studied by peak-differentiating and imitating analysis. The divided peaks are located at 199.0 and 197.4 eV for Cl 2p_1/2_ and Cl 2p_3/2_, respectively (Fig. 4d). The XPS results further confirm that the formation of Ag/Au/AgCl hollow heterostructures.

### Optical Property of Ag NWs and Ag/Au/AgCl Hollow Heterostructures

**Fig. 5:**
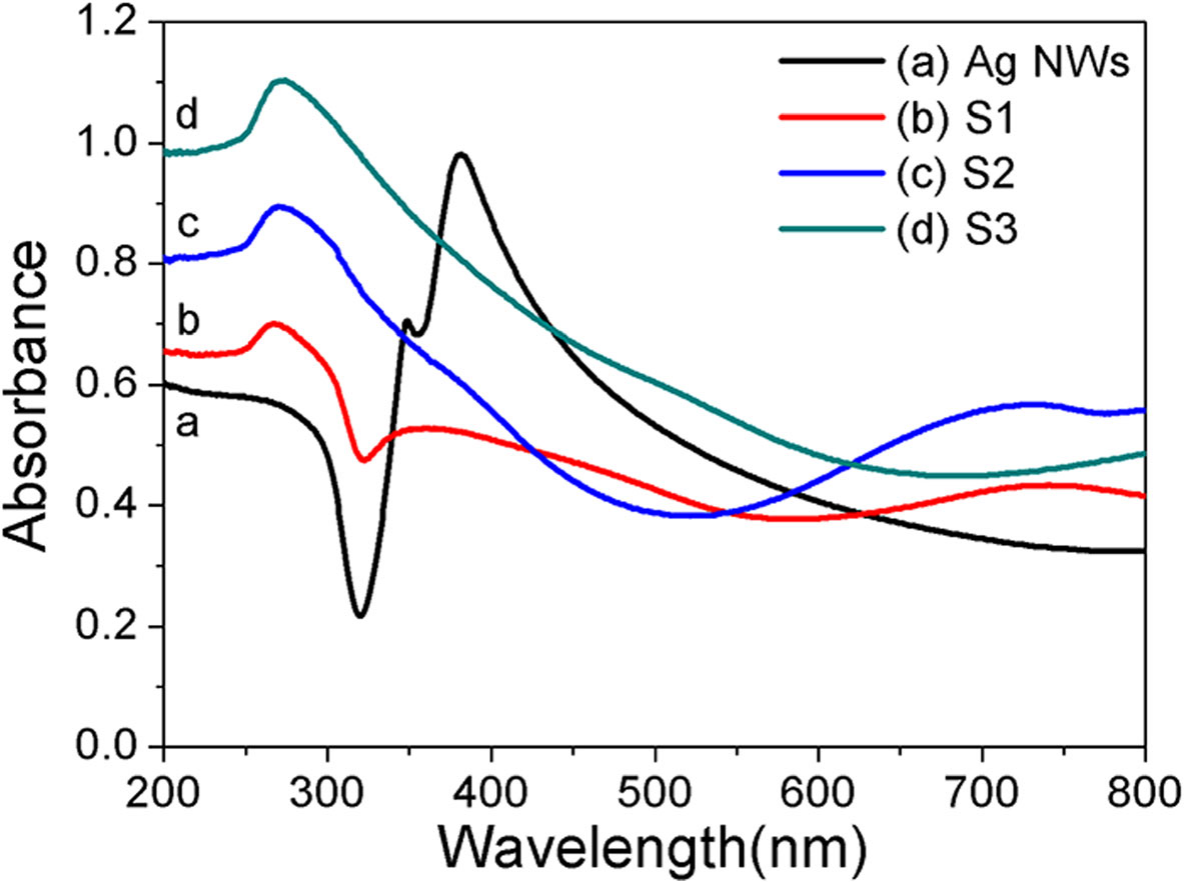
The UV-vis absorption spectra of Ag NWs and Ag/Au/AgCl sample S1–S3

Before the catalytic test, the UV-vis absorption spectra of Ag NWs and Ag/Au/AgCl (S1-S3) are presented in Fig. 5. The main absorption peak of Ag NWs is located at ~ 380 nm (black line) due to the transverse plasmon mode of Ag NWs [29]. Otherwise, the appearance of a shoulder absorption peak at ~ 350 nm is presumably due to plasmon response of the long silver NWs, which is similar to that of bulk silver [30]. As shown in the UV-vis absorption spectra of Ag/Au/AgCl (S1–S3), the positions and intensities of these absorption peaks are altered gradually. That is, the absorption intensity of characteristic peak of Ag NWs (~ 380 nm) is decreased gradually. While a new absorption peak located at 280 nm, as a characteristic absorption peak of AgCl is presented in S1, S2, and S3. Moreover, broad peaks at the range of 600 ~ 800 nm in S1 and S2 are noticed; it could be attributed to LSPR absorption peaks of metallic Au. But this absorption intensity is decreased in S3, because the Ag/Au bimetals are coated by AgCl particles gradually with the addition of HAuCl_4_ solution. This result further confirms the conjectures of SEM images in Additional file 1: Figure S1c and S1d.

### Catalytic Performance of Ag/Au/AgCl Hollow Heterostructures

**Fig. 6:**
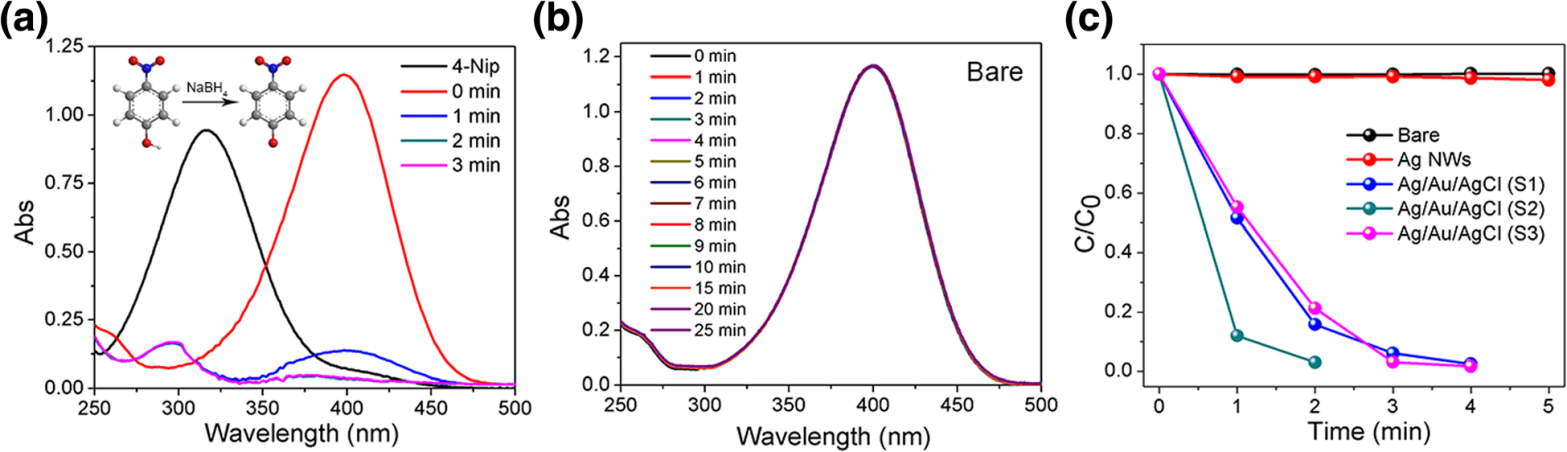
**a** UV-vis spectral changes of Nip by using 0.025 mg of S2 as catalyst. **b** Time-dependent UV-vis spectrum of Nip without any catalysts. **c** Normalized concentration change of Nip in the presence of 0.025 mg as-prepared samples

Noble metal is a favorable candidate for reduction of Nip to Amp through catalysis. The resulted Amp is an intermediate and raw material for fine chemicals, such as pharmaceuticals, pesticides, and dyes. Thus, in this section, a heterostructure composed of two noble metals and one semiconductor is fabricated for reduction of Nip to Amp. A typical adsorption peak of Nip located at 317 nm is presented when using 0.025 mg of S2 as catalyst (Fig. 6a). While this absorption peak is shifted to the wavelenght of 400 nm after adding NaBH_4_ solution [31]. However, this absorption peak located at 400 nm disappeared within 2 min, and a new absorption peak emerged at 295 nm is generated, indicating the generation of Amp. Theoretically, this catalytic process follows the pseudo-first-order reaction [32, 33]:

$$ -\ln \left(C/{C}_0\right)= kt $$

Where *C*_0_ and *C* are the original concentration and the constant concentrations of the Nip or dye solution, respectively. *k* is the apparent rate constant of the catalytic reaction, then all the *k*_1_ values during catalytic reaction are shown in Table 1. The control test shows that no obvious degradation is observed without addition of catalysts (Fig. 6b), the apparent rate constant (*k*_1_) value of this self-degradation process is 0.0466 × 10^−2^ min^−1^. The transfer rates of Nip to Amp with time evolution over 0.025 mg of these as-prepared samples (Ag NWs and S1-S3) are exhibited in Fig. 6c. 0.025 mg of Ag NWs shows no obvious catalytic performance for the reduction of Nip (Additional file 1: Figure S2a). But, when increasing the amount of Ag NWs to 0.1 mg, significant catalytic performance for Nip transference over time is presented. After reaction for 15 min, about 83.6% of Nip is reduced into Amp, demonstrating that the Ag NWs still show catalytic activity in reduction of Nip (Additional file 1: Figure S2b). Comparatively, sample S1–S3 show higher catalytic activities in this reduction process when using 0.025 mg of S1, S2 and S3 (Additional file 1: Figure S3a**,** Fig. 6 and Figure S3b). The *k*_1_ values of S1–S3 are calculated as 123 × 10^−2^ min^−1^, 177 × 10^−2^ min^−1^, and 111 × 10^−2^ min^−1^, respectively. Apparently, the Ag/Au/AgCl hollow heterostructures show enhanced catalytic performances than pure Ag NWs. It is probably due to the as-generated Ag/Au bimetal could promote electrons transfer [17]. On the other hand, the loose surfaces and hollow structures of Ag/Au/AgCl heterostructures provide more active sites than Ag NWs for reaction.

**Fig. 7:**
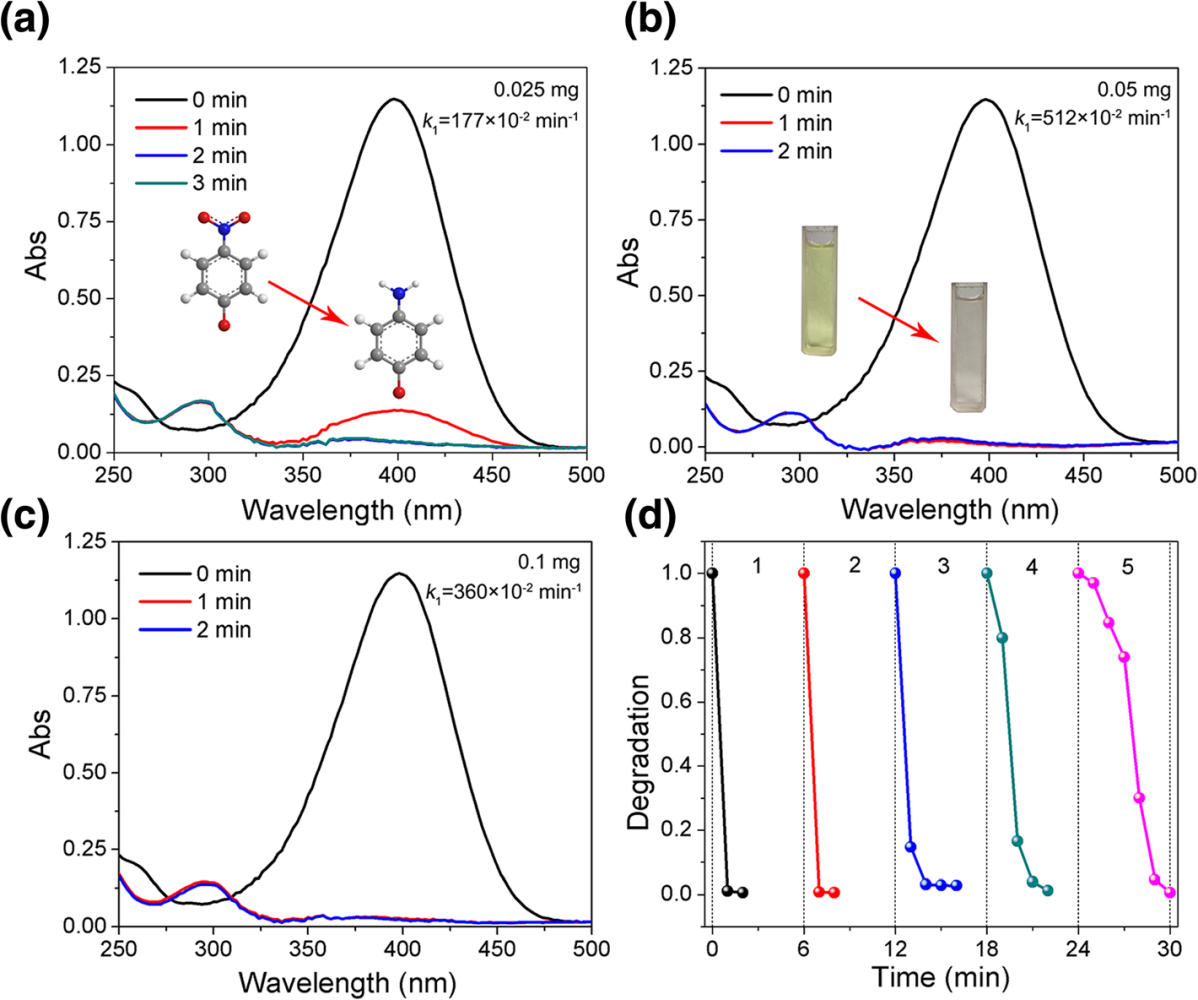
Time-dependent UV-vis spectrum and corresponding *k*_1_ values of Nip with **a** 0.025 mg, **b** 0.05 mg, and **c** 0.1 mg of S2; the inset in **a** is the diagram for reaction of Nip to Amp, and the inset in **b** is the color change of Nip solution after catalytic reaction. **d** Normalized concentration change of Nip for 5 cycles in the presence of 0.05 mg of S2

Additionally, the catalytic performances of S2 with different amount (0.025 mg, 0.05 mg, and 0.1 mg) are also tested (Fig. 7a–c). All these absorbances of Nip are decreased promptly; it means that the Nip could be reduced into Amp in 2 min. The *k*_1_ values of these reactions are 177 × 10^−2^ min^−1^, 512 × 10^−2^ min^−1^, and 360 × 10^−2^ min^−1^, respectively. The inset in Fig. 7a is the diagram for reaction of Nip to Amp, the color change of Nip solution after catalytic reaction with the presence of S2 in Fig. 7b also confirms the catalytic reaction. Moreover, the recycled catalytic property of Ag/Au/AgCl (S2) is also investigated. As shown in Fig. 7d, after five times of reaction cycles, the complete reduction of Nip still can be achieved within 6 min, indicating the good catalytic activity of Ag/Au/AgCl (S2) sample. Here, the reaction time of S2 in this catalytic reaction is increased from 1 min to 6 min, it is probably because the catalyst is too few to full recycle, mass loss of the sample during the centrifugation and transfer process is unavoidable; thus, the catalytic activity is decreased gradually.

### Photocatalytic Performances of Ag/Au/AgCl Hollow Heterostructures

**Fig. 8:**
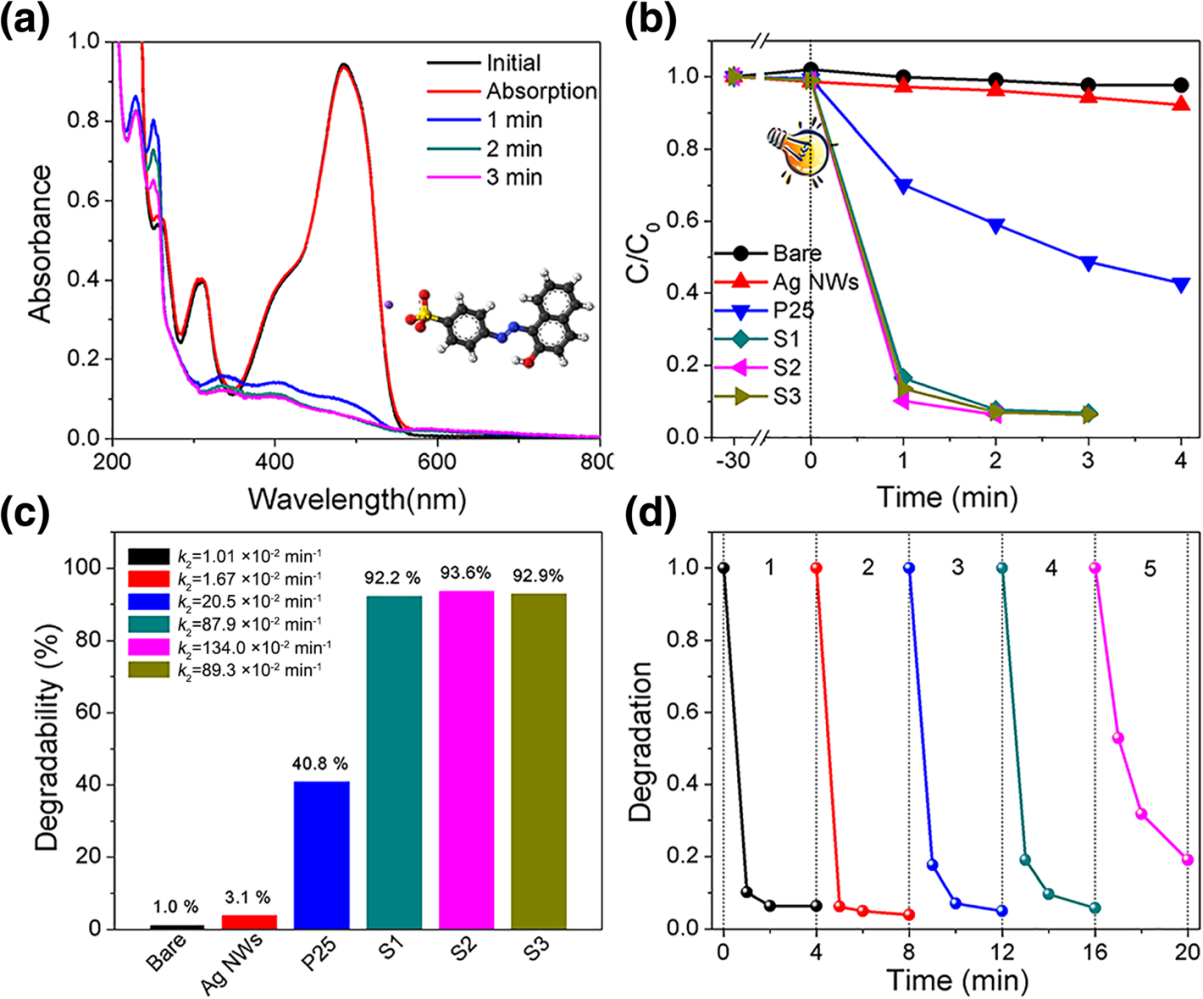
**a** Time-dependent UV-vis spectrum of AO7 with the presence of S2 (inset is 3D molecular formulas of AO7). **b** Normalized concentration change of AO7 with 3 mg of as-prepared samples. **c** The degradability of AO7 with as-prepared samples after light illumination for 2 min. **d** The recycled photodegrading of the AO7 over the S2 for five times

Photocatalytic performance is another index to test the catalytic activities of these Ag/Au/AgCl hollow heterostructures. In this experiment, the photocatalytic performances of Ag NWs, Ag/Au/AgCl samples (S1–S3), and P25 are characterized by degrading the organic dyes of AO7 under simulated sunlight illumination. According to the typical time-dependent UV-vis spectrum of AO7 with the presence of S2 (light illumination interval is 1 min), about 93.6% of AO7 are degraded in 2 min (Fig. 8a). The normalized concentration changes of AO7 with the presence of these catalysts (Fig. 8b). Evidently, Ag NWs show no obvious photocatalytic performance for degradation of AO7, because noble metal could only act as cocatalyst in photocatalytic process. P25 show some photocatalytic activity in degradation of AO7, due to the UV light absorption. Clearly, the photocatalytic performances of all Ag/Au/AgCl (S1–S3) samples are improved than pure Ag NWs and P25. It is probably because the coupling of Au/Ag bimetal with AgCl could promote the separation of charges. The degradability and corresponding *k*_2_ value of AO7 with as-prepared samples are presented in Fig. 8c. The degradation rates of blank sample, Ag NWs, and P25 are 1%, 3%, and 40.8%, respectively. The Ag/Au/AgCl (S1–S3) samples show enhanced degradation rates (92.2% for S1, 93.6% for S2, and 92.9% for S3) than Ag NWs and P25 after light illumination for only 2 min. The *k*_2_ values of Ag NWs and P25 are 1.67 × 10^−2^ min^−1^ and 20.5 × 10^−2^ min^− 1^, respectively, and these values of S1–S3 improved to 87.9 × 10^−2^, 134.0 × 10^−2^, and 89.7 × 10^−2^ min^−1^, respectively (Fig. 8c and Table 1). It demonstrates the high performance of Ag/Au/AgCl hollow heterostructures. The photocatalytic stability of Ag/Au/AgCl (S2) hollow heterostructures is further investigated, and the result is shown in Fig. 8d. This sample present for five recycling times, more than 80% of dyes are degraded in 4 min, indicates a good photocatalytic stability of Ag/Au/AgCl (S2) hollow heterostructures under simulated solar light illumination. A slight decrease of the photocatalytic property could be attributed to the AgCl decomposition and mass loss during the centrifugation and transfer process.

### Proposed Enhanced Catalytic and Photocatalytic Mechanisms

**Fig. 9:**
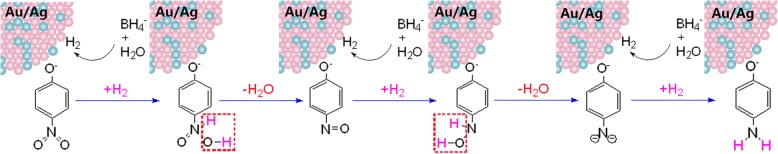
The catalytic mechanism proposed for Nip reduction to Amp by NaBH_4_ on the surface of Ag/Au bimetals

(a) The enhanced catalytic performance of these Ag/Au/AgCl NWs could be attributed to the synergistic catalysis of Ag/Au bimetals. This reduction reaction will not happen without catalysts, because both Nip and BH^4−^ are electronegative and the kinetic barrier between them is extremely high. After the catalyst adding, the kinetic barrier is low. [34] Because charged surface of Ag/Au bimetals is facilitated to adsorbing ionic reactants and forming an activity site for electron transfer. During the specific reduction process of Nip, two processes including hydrogen producing and consuming are involved in this reaction (Fig. 9). Firstly, the hydrogens are produced by oxidation of BH_4_^−^. Then, these hydrogen species could participate in subsequent additive reaction of –N=O group. After dehydration process, the as-obtained nitrosobenzene could further react with hydrogen species for generation of N-phenylhydroxylamine. Ultimately, the Amp is finally obtained by multiple addition reaction and dehydration reaction [35, 36]. During these reaction processes, noble metal Au and Ag could act as mediums for charge transfer. More significantly, in this catalyst, the Ag/Au bimetals could enhance the fundamental catalytic activity. Because the work function of Ag and Au is different. Electrons could transfer from Ag to Au because the Fermi level of Ag is lower than Au (The detail information could be further discussed in next section.). As a result, the electrons surplus area and electrons depletion area could be formed in Au and Ag, respectively. After adsorbing ions (BH_4_^−^ and Nip^−^), the Ag/Au bimetals act as medium for uptaking electrons from BH_4_^−^ and releasing electrons to Nip. Both electrons surplus area and electrons depletion area are increased when more interfaces between Au and Ag are emerged. These areas could also increase the chances for absorption of the ions (BH_4_^−^ and Nip^−^). Consequently, the interface between Au and Ag helps the noble metal to act as an electron relay system in this reaction. The function of these Ag/Au bimetals provides the pathways to understand the intermediate steps in reaction of adsorbed species.

As to the different samples, firstly, the Ag NWs show a low catalytic activity in reduction of Nip, it probably because these Ag NWs with smooth surface lack activity site for reaction. For these Ag/Au/AgCl NWs samples, the Ag/Au bimetals are responsible for the reduction of Nip, and the AgCl acts as support material. With the increase of Au and AgCl, the alloying of Au/Ag is improved, and the surface of Ag/Au/AgCl NWs is rougher. As described above, the catalytic activities could be improved gradually. However, in the catalytic results, S2 sample shows the best catalytic performance among all samples. The increased AgCl could influence the catalytic activities of Ag/Au bimetals. The excess AgCl is coated on the surface of Ag/Au bimetals in S3 sample, and it prevents the contract of Au/Ag with Nip. This result could be conformed with the SEM images and UV-vis spectrum of S3 sample.

**Fig. 10:**
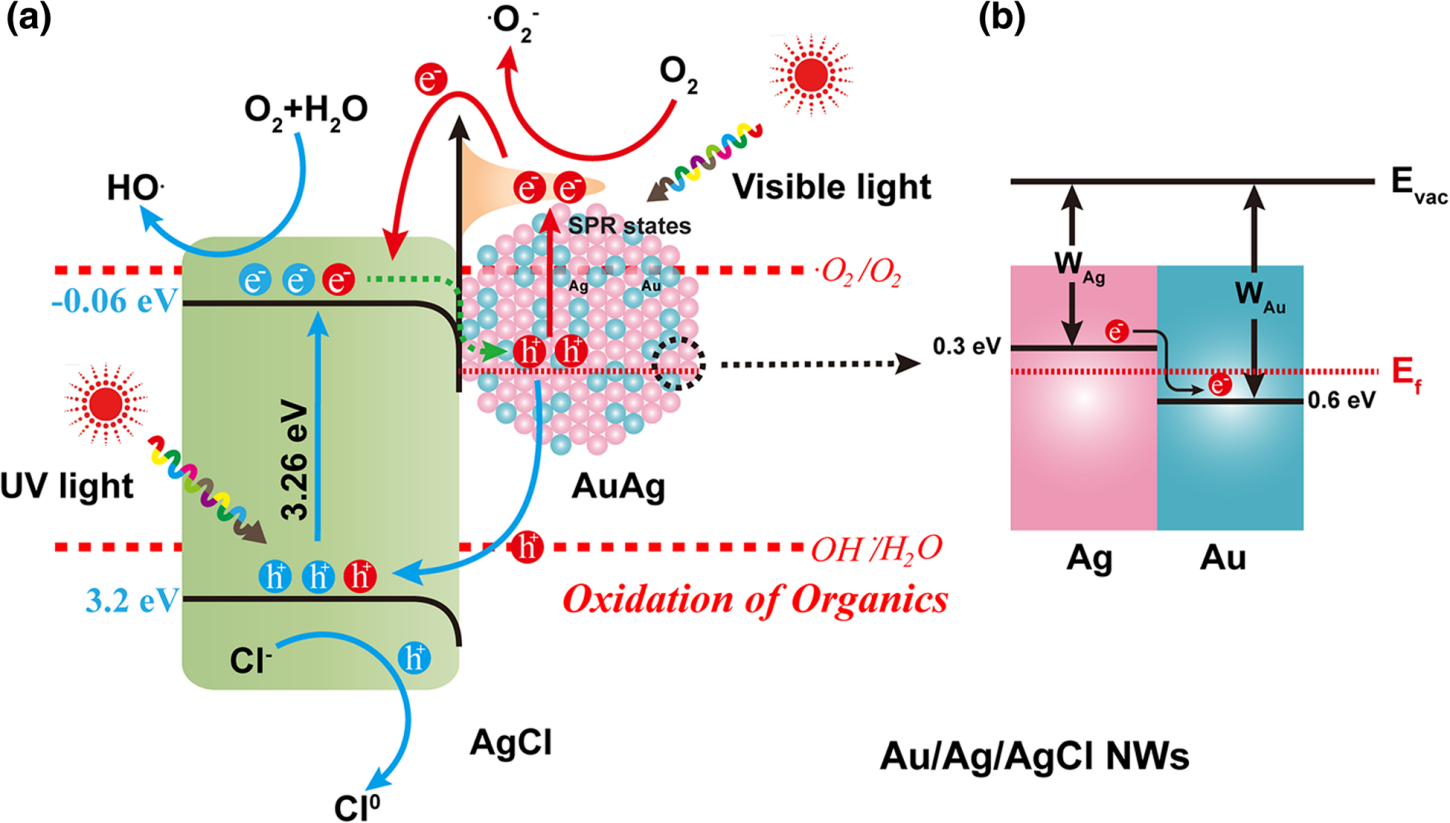
The proposed photocatalytic mechanism of Ag/Au/AgCl hollow NWs. **a** Schematic diagram of charge transfer. **b** Electron transfer in Au/Ag

(b) The enhanced photocatalytic performance of these Ag/Au/AgCl hollow NWs could be attributed to the synergistic effect of the LSPR and PRET mechanisms. Figure 10a shows the proposed enhanced photocatalytic mechanism of these composited heterostructures. The bandgap of AgCl is 3.26 eV, and the position of conductive band (CB) and valence band (VB) are − 0.06 eV and 3.2 eV, respectively [37]. The CB is lower than the ${E}_{O_2/.{O_2}^{-}}$ of O_2_/·O_2_^−^ (− 0.28 eV, on the normal hydrogen electrode (NHE) scale). The electrons on CB of AgCl could not be captured by O_2_ for O_2_^−^. But the VB of AgCl is lower than ${E}_{\mathrm{OH}/{\mathrm{H}}_2\mathrm{O}}$of ·OH/H_2_O (2.27 eV on NHE scale), holes on the VB could be captured by water for producing of ·OH. The work function of Au and Ag are 5.1 and 4.8 eV, respectively. The positions are 0.6 and 0.3 eV on the NHE scale. The Fermi level of Ag is higher than Au, the electrons could transfer from Ag to Au for a new Fermi balance (*E*_*f*_) when they contact with each other (Fig. 10b), and the built-in electric field on the interface of Au and Ag could promote charge separation [38, 39]. Simultaneously, another new balance is reached when the Au/Ag contact with AgCl.

Theoretically, the photocatalytic performances of semiconductors could be improved by coupling with the noble metals. The LSPR effect of noble metals promotes the separation of charges [40]. The SPR electrons jump to the CB of semiconductors and participate in reduction reaction, and the oxidation reaction happen with the holes left on noble metal [41]. However, there still have some electrons that are retreated for recombination with holes. In addition, the excess inpouring electrons could not participate in reaction timely, and they recombine with holes of semiconductors. These two phenomena are the important factors for reducing the photocatalytic performances [42]. Thus, a moderate amount of noble metal is facilitated to improve the photocatalytic activities of semiconductor. By comparing with a single metal (such as Ag), bimetal (such as Ag/Au alloy) induced to semiconductor could not only broaden the SPR absorption, but also promote the transfer of charges. The electrons depletion area caused by Fermi level difference in Ag could promote the oxidation reaction on the surface of Ag metal [18]. Furthermore, when the LSPR wavelength matches well with the energy levels of the semiconductor, the LSPR energy could transfer to semiconductor; this phenomenon is called as plasmon resonance energy transfer (PRET). This PRET effect can enlarge the electromagnetic field intensity of incoming light for promoting the formation of electron/hole pairs [43]. Two LSPR frequencies of Ag/Au bimetals can match two different semiconductors; thus, the photocatalytic performance could be improved further in some degree. In the specific photocatalytic process, AgCl can only absorb UV light due to its intrinsic wide band gap. The photo-generated electrons are captured by H_2_O and O_2_ for ·OH. The left holes are combined with Cl^−^ on the surface of AgCl, and the Cl^0^ with strong oxidability is obtained [44]. On the other hand, the Ag/Au bimetals which produced the SPR electrons could be captured by O_2_ for ·O_2_^−^, and another part of electrons are transferred to the CB of AgCl. A part of these electrons retreats to Au/Ag, causing the reduction of photocatalytic activities. The hole left on Ag/Au bimetals could partake in the synthesis of Cl^0^. The ·OH, ·O_2_^−^, and Cl^0^ show the high activities for degradation of organic pollutants. Thus, photocatalytic performance of Ag/Au/AgCl hollow NWs is better than Ag NWs and P25.

However, as to these three groups of Ag/Au/AgCl hollow NWs, the different photocatalytic performances are decided by the synergetic activities of semiconductors and noble metals. In this Ag/Au/AgCl hollow NWs, the different ratio of Au, Ag, and AgCl could influence the production and separation effective of charges. Specifically, the total number of noble metal atoms (Au and Ag) is reduced gradually from S1 to S3. The LSPR effect of Au and Ag is reduced correspondingly. According to the aforementioned theory, excess amount of noble metal is not beneficial to improve the photocatalytic activity of semiconductor, because excess SPR electrons can consume the holes on VB of AgCl. In addition, when the ratio of Au and Ag is increased gradually, in order to reach the new balance of Fermi level, the Au is more electronegative and Ag is more electropositive. This electropositive surface of Ag is propitious to form a Cl^0^ with Cl^−^. Thus, the large ratio of Au and Ag could improve the photocatalytic performance. On the other hand, the absorption range of Ag NWs is 320 ~ 600 nm, and the theoretical absorption band of AgCl is 380 nm. The PRET effect of Ag could partially match with the band gap of AgCl, it promotes the AgCl to produce more electron/hole pairs. More metal Ag in these Ag/Au/AgCl hollow NWs is more favorable for photocatalytic reaction. But the influences of former two factors are greater than the third one. Moreover, AgCl as a wide bandgap semiconductor could absorb UV light for effective UV light-driven photocatalytic performance. The content of AgCl in samples S1, S2, and S3 is increased gradually, and the photocatalytic activities are also increased in theory. While the AgCl not only acts as photocatalyst under UV light, it also provides a carrier for charge separation and provides an electron donator for the oxidation of Cl^−^ and the holes in noble metal. Thus, more AgCl in these Ag/Au/AgCl hollow NWs are more favorable. After the comprehensive analysis of the two systems (Ag/Au bimetals and AgCl semiconductor), it proposes that the photocatalytic performances of S1, S2, and S3 should be improved gradually. But in fact, S2 shows the best photocatalytic activity, because morphology is another important factor to influence the photocatalytic performances. The increased amount of AgCl in S3 could encapsulate the Ag/Au bimetals, and it prevents most of light absorption of noble metal. This proclaim can be confirmed by the SEM images (Additional file 1: Figure S1c and S1d) and UV-vis spectrum (Fig. 5). Additionally, the increased Au atoms could also prevent the Ag contact with Cl^−^. And the photocatalytic activity of AgCl under UV light cannot be neglected. Thus, the sequence for photocatalytic performance of these samples is S2 > S3 > S1.

## Conclusions

In summary, 1D Ag/Au/AgCl hollow heterostructures are successfully synthesized by GRR method from Ag NWs. These as-obtained Ag/Au/AgCl hollow heterostructures show the high catalytic performance than pure Ag NWs by reducing Nip into Amp, and the AgCl semiconductor could act as supporting materials, but the excess AgCl is the obstacle for contact of Ag/Au bimetals with reactive species. Moreover, Ag/Au/AgCl hollow heterostructures also present the excellent photocatalytic performances than pure Ag NWs and commercial P25, and the Ag/Au bimetals enhanced the photocatalytic activity of AgCl semiconductor via the LSPR and PRET mechanisms. These synthesized 1D Ag/Au/AgCl hollow heterostructures with multifunction could provide favorable route for preparing other noble metal-based composite catalysts.

## Additional files

Additional file 1: **Figure S1.** (a) SEM image of Ag/Au/AgCl hollow heterostructures (S2). (b) The corresponding magnified SEM image, (c) SEM image of Ag/Au/AgCl hollow heterostructures (S3). (d) The corresponding magnified SEM image. **Figure S2.** Time-dependent UV-vis spectrum of Nip with (a) 0.025 mg and (b) 0.1 mg of Ag NWs. **Figure S3.** Time-dependent UV-vis spectrum of Nip with (a) 0.025 mg of S1 and (b) 0.025 mg of S3. (DOCX 1439 kb)

## Abbreviations

0D: Zero-dimensional
1D: One-dimensional
2D: Two-dimensional
3D: Three-dimensional
Amp: 4-aminophenol
AO7: Acid orange 7
CB: Conductive band
GRR: Galvanic replacement reaction
HRTEM: High-resolution transmission electron microscopy
LSPR: Localized surface plasmon resonance
NHE: Normal hydrogen electrode
Nip: 4-Nitrophenol
NPs: Nanoparticles
NWs: Nanowires
PRET: Plasmon resonance energy transfer
PVP: Polyvinylpyrrolidone
SEM: Scanning electron microscopy
TEM: Transmission electron microscopy
UV: Ultraviolet
VB: Valence band
XPS: X-ray photoelectron spectroscopy
XRD: Powder X-ray diffraction
