# Supplementary material for: Catalytic Application and Mechanism Studies of Argentic Chloride Coupled Ag/Au Hollow Heterostructures: Considering the Interface Between Ag/Au Bimetals
Source: Nanoscale Res Lett. 2019 Jan 25;14:35. doi: 10.1186/s11671-019-2862-9 (PMC6349269; doi:10.1186/s11671-019-2862-9)
Supplement: Supplementary file 1 — Figure S1. (a) SEM image of Ag/Au/AgCl hollow heterostructures (S2). (b) The corresponding magnified SEM image, (c) SEM image of Ag/Au/AgCl hollow heterostructures (S3). (d) The corresponding magnified SEM image. Figure S2. Time-dependent UV-vis spectrum of Nip with (a) 0.025 mg and (b) 0.1 mg of Ag NWs. Figure S3. Time-dependent UV-vis spectrum of Nip with (a) 0.025 mg of S1 and (b) 0.025 mg of S3. (DOCX 1439 kb) [file 11671_2019_2862_MOESM1_ESM.docx]

***Supporting Information*** for

Catalytic Application and Mechanism Studies of Argentic Chloride Coupled Ag/Au Hollow Heterostructures: Considering the Interface Between Ag/Au Bimetals

*Jun Liu^1, 3, 4 #^, Zhaohui Wu^2, 3 #^, Quanguo He^1^, Qingyong Tian^3, 4^, Wei Wu^3^ ^[[1]](#footnote-1)^*, Xiangheng Xiao^4^ *, Changzhong Jiang^4^ **

^1^ Hunan Key Laboratory of Biomedical Nanomaterials and Devices, Hunan University of Technology, Zhuzhou 412007, P. R. China

^2^ Hunan Key Laboratory of Applied Environmental Photocatalysis, Changsha University, Changsha 410022, P. R. China

^3^ Laboratory of Printable Functional Nanomaterials and Printed Electronics, School of Printing and Packaging, Wuhan University, Wuhan 430072, P. R. China

^4^ Key Laboratory of Artificial Micro- and Nano-structures of Ministry of Education, School of Physics and Technology, Wuhan University, Wuhan 430072, P. R. China

^#^ These authors contributed equally.


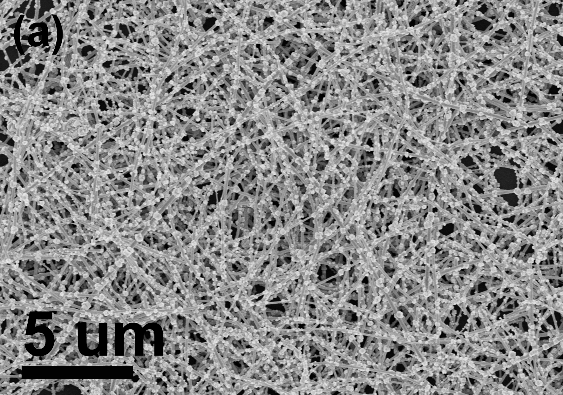

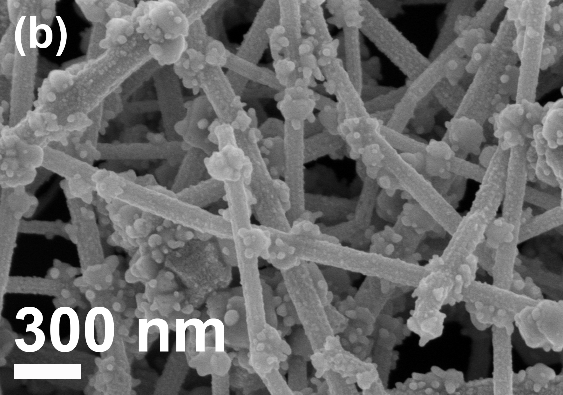


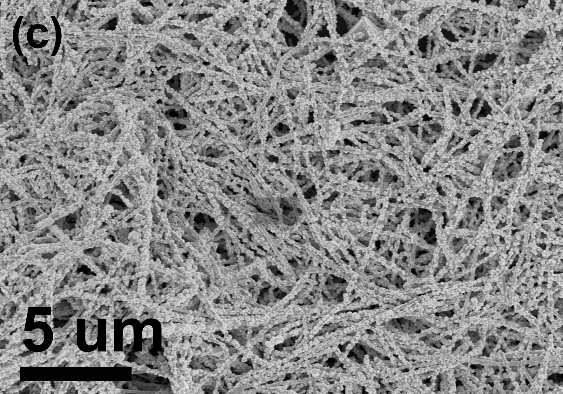

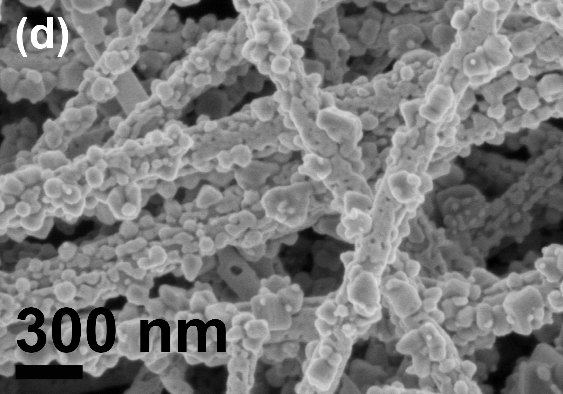


Figure S1. (a) SEM image of Ag/Au/AgCl hollow heterostructures (S2), (b) The corresponding magnified SEM image, (c) SEM image of Ag/Au/AgCl hollow heterostructures (S3), (d) The corresponding magnified SEM image.


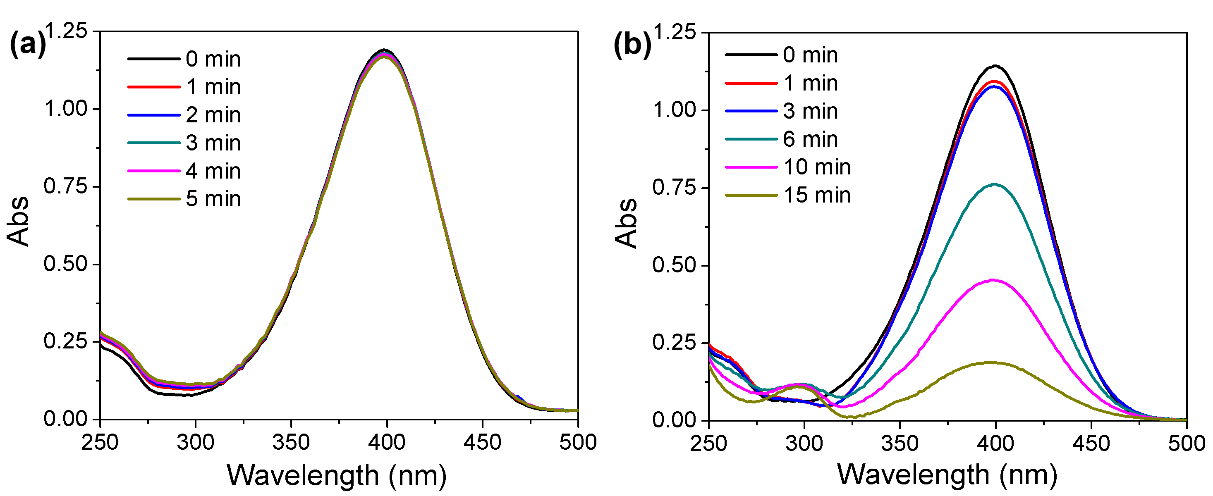


Figure S2. Time-dependent UV-vis spectrum of Nip with (a) 0.025 mg and (b) 0.1 mg of Ag NWs.


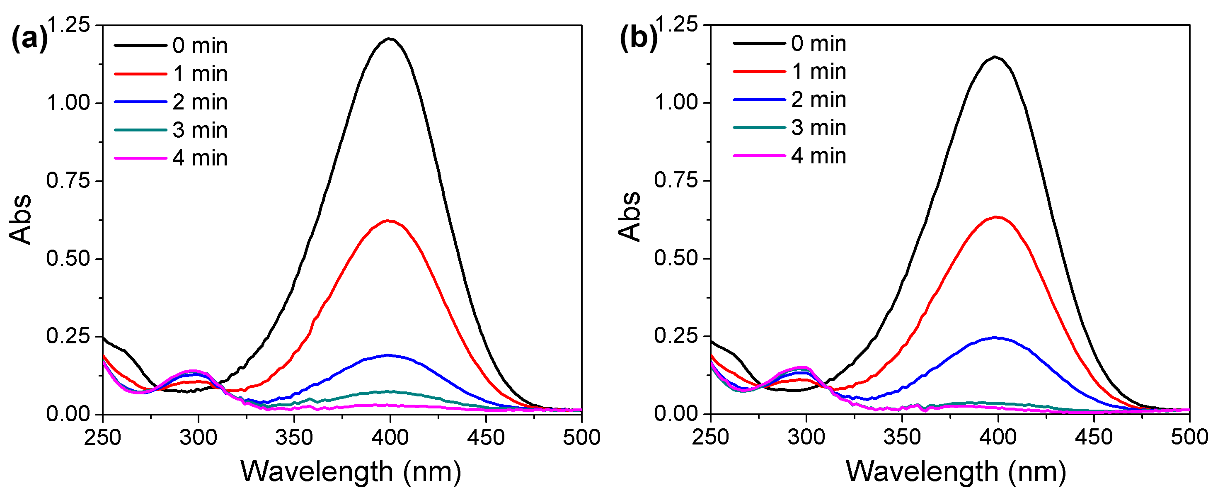


Figure S3. Time-dependent UV-vis spectrum of Nip with (a) 0.025 mg of S1 and (b) 0.025 mg S3.

1. *To whom correspondence should be addressed. Tel: +86-27-68778529. Fax: +86-27-68778433. E-mail: [weiwu@whu.edu.cn](mailto:weiwu@whu.edu.cn) (W. Wu), [xxh@whu.edu.cn](mailto:xxh@whu.edu.cn) (X.H. Xiao), [↑](#footnote-ref-1)
